# Artificial Intelligence for Radiographic Image Quality: Radiographers at the Forefront

**DOI:** 10.1002/jmrs.70050

**Published:** 2025-12-12

**Authors:** Kamarul Amin Abdullah

**Affiliations:** ^1^ School of Medical Imaging, Faculty of Health Sciences Universiti Sultan Zainal Abidin Kuala Terengganu Terengganu Malaysia

## Abstract

This editorial highlights the central role of radiographers in leading AI‐driven radiographic image‐quality assessment. It outlines how AI can enhance real‐time feedback, support consistency, and strengthen safe, patient‐centered imaging practice.

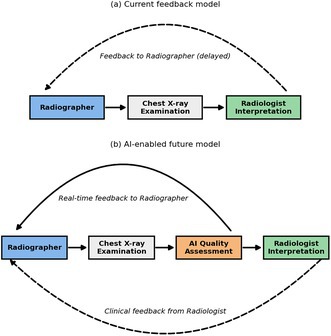

## 1 Introduction

Artificial intelligence (AI) is redefining radiographic image quality, and radiographers are at the forefront of that change. From diagnostic decision support to workflow automation, AI is transforming how we acquire, assess, and interpret medical images. In medical imaging, AI's most promising applications have centered on disease detection and segmentation [1]. For example, AI models have been successfully applied to automatically segment the left ventricular myocardium in cardiac CT scans, achieving high accuracy and demonstrating potential for early disease detection [2]. However, the integration of AI into image quality assessment, particularly in general radiography, represents a vital yet underexplored frontier. This is especially relevant to radiographers, who are responsible for image acquisition and quality assurance, as well as for ensuring patient safety and diagnostic value.

**FIGURE 1:**
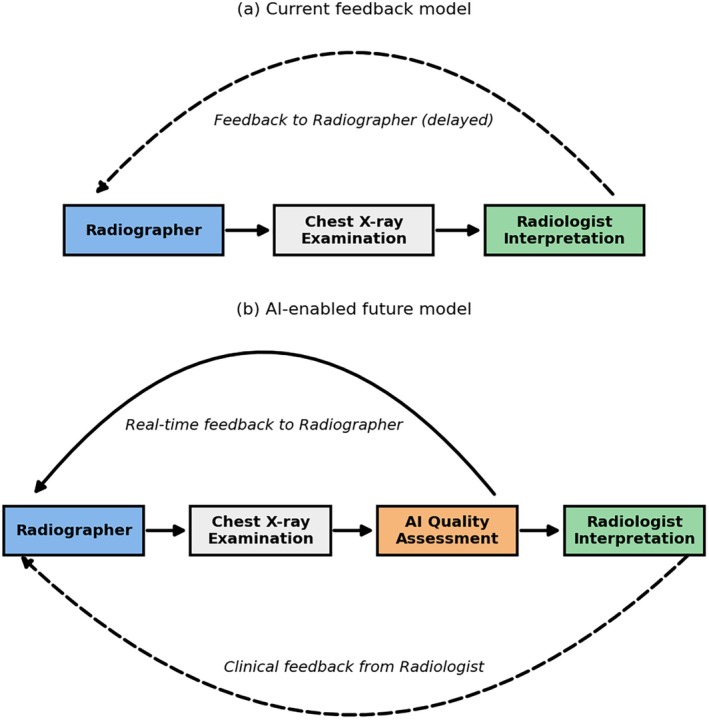
Comparison of radiographic image‐quality feedback models. (a) Current feedback model: Feedback to radiographers typically occurs immediately after image acquisition through their own technical evaluation, with additional feedback from radiologists when images are deemed clinically suboptimal. (b) Artificial intelligence provides real‐time image‐quality feedback directly to radiographers at the point of acquisition, while radiologist interpretation continues to provide clinical guidance when required.

Chest radiography, being one of the most performed examinations worldwide, including across Australia and New Zealand, is particularly prone to suboptimal acquisitions [3], often due to positioning errors, motion artefacts, or poor exposure [4]. These issues not only compromise diagnostic accuracy but also increase the need for repeat imaging, thereby raising patient radiation exposure and delaying care. The need for efficient, consistent, and scalable image quality assessment tools is therefore urgent. As radiographers strive for precision under increasing workload and time pressures, AI‐driven systems may offer real‐time support and constructive feedback. Integrating such tools into routine practice could enhance radiographers' professional judgement, standardise image quality evaluation, and reduce variability across practitioners and institutions. Figure 1 compares the existing radiologist‐led feedback workflow with a proposed AI‐enabled model that delivers real‐time quality feedback directly to radiographers at the point of acquisition.

In current practice, radiographers already make immediate technical judgments on image adequacy, particularly for evident issues such as under‐ or over‐exposure and motion artefacts, while radiologist feedback is typically sought only when additional diagnostic input is required. AI can further enhance this process by providing objective, standardized feedback and reducing uncertainty in borderline or complex cases, thereby promoting confidence, consistency, and efficiency in clinical decision‐making.

## 2 Contextualising the Featured Study

In this issue of the Journal of Medical Radiation Sciences, Liu et al. [5] present a timely and significant contribution to the growing body of work at the intersection of AI and radiographic image quality. Their study evaluates the performance of two convolutional neural networks (CNNs), namely DenseNet121 and YOLOv8, in classifying suboptimal chest radiographs using a large, diverse dataset comprising posteroanterior (PA), lateral, and even paediatric chest X‐rays. Here, suboptimal refers to images with technical deficiencies that compromise diagnostic quality. What sets this study apart is not only its methodological robustness but also its relevance to daily clinical radiographic practice. Suboptimal chest radiographs are a well‐documented challenge, often resulting in unnecessary repeat exposures and diagnostic delays. Yet, until now, few AI studies [6, 7, 8] have tackled the issue of technical quality evaluation in general radiography with real‐world relevance. This study does exactly that, bridging the gap between technical innovation and clinical need.

DenseNet121 and YOLOv8 demonstrated excellent diagnostic performance (AUROC: 0.97 and 0.95, respectively), with DenseNet121 slightly outperforming YOLOv8. Beyond AUROC, future studies should report sensitivity and specificity at clinically relevant decision thresholds. These results are more than numbers; they signal a future where radiographers may be supported by intelligent tools capable of identifying technical deficiencies such as clipped anatomy, motion blur, or poor positioning before a radiologist even views the image. Importantly, the authors incorporated radiographer expertise into their ground truth dataset, reflecting the profession's central role in defining what constitutes image quality. They also included a comparison against radiologists' assessments, acknowledging the interplay between radiographer technical judgment and radiologist diagnostic expectations, which is an essential dynamic in radiographic quality assurance.

By focusing on image quality rather than disease detection, this work highlights the unique value radiographers bring to the diagnostic process and positions AI as a collaborator, not a competitor, in improving radiographic practice. Nevertheless, while the study demonstrates promising diagnostic performance, it is important to acknowledge that its generalisability may be influenced by dataset representativeness and limited external validation. Future research should explore model performance across multi‐centre datasets and varying acquisition conditions to ensure clinical robustness.

## 3 Clinical Implications for Radiographers

The featured study arrives at a pivotal time for the radiography profession. As healthcare systems increasingly embrace digital transformation, radiographers are expected to evolve beyond their traditional roles and actively participate in the design, adoption, and evaluation of AI technologies. One of the key takeaways from this study is the potential for AI to act as a real‐time decision support tool, particularly for junior radiographers, interns, and students. The integration of CNNs into the radiographic workflow, especially at the image acquisition stage, could help radiographers identify suboptimal images before they reach the Picture Archiving and Communication System (PACS). This not only enhances diagnostic accuracy but also promotes confidence and independence in clinical decision‐making. Operational value should be tracked using the reject–repeat rate, the proportion of suboptimal images, time‐to‐report, reader concordance, and dose per study.

In addition, the inclusion of both paediatric and lateral chest X‐rays in the dataset makes the model more reflective of real‐world practice where complexity, variability, and patient cooperation often challenge even experienced practitioners. For radiographers, this signifies that AI tools are being developed with clinical nuance in mind, rather than as abstract or overly controlled research experiments.

Importantly, this study reinforces the need for radiographer‐led involvement in the development and implementation of AI systems. From annotating datasets to co‐designing feedback interfaces, radiographers must be embedded in every stage of AI translation. This ensures that algorithms align with practical realities, uphold patient safety standards, and respect professional judgment.

Implementing AI‐driven image quality tools in diverse clinical environments, such as low‐resource, rural, or after‐hours settings, presents unique challenges. Limited infrastructure, connectivity issues, and variable technical support may hinder seamless integration. Collaborative efforts between professional bodies, technology providers, and healthcare administrators are essential to ensure that AI benefits are equitably distributed across all practice settings.

Nevertheless, to fully realise this potential, radiographers must be equipped with AI literacy and digital competencies. Training programmes and continuous professional development (CPD) frameworks should now include fundamental knowledge in AI, data interpretation, and algorithmic bias. AI readiness is no longer optional; it is essential for safe and effective integration of technology into radiographic practice. In this context, the study not only advances the science, but it also raises the bar for what radiographers should expect from AI in everyday practice.

## 4 Way Forward: A Role Shift

These findings represent more than a proof of concept; they point toward a paradigm shift in how image quality could be managed, audited, and improved in clinical radiography. As AI systems become increasingly sophisticated, future developments should aim to go beyond binary classification (optimal vs. suboptimal) toward identifying specific technical deficiencies such as poor inspiration, clipped anatomy, rotation, or artefacts. AI quality assessment must operate under human‐in‐the‐loop governance, with routine drift monitoring and bias checks across age groups, body habitus, and equipment vendors, co‐led by radiographers. This level of granularity would offer targeted feedback, which could be integrated into quality assurance dashboards or even directly at the imaging console to guide corrective actions in real‐time.

Nevertheless, to ensure these tools are clinically meaningful, they must be co‐developed with radiographers, radiologists, medical physicists, and AI specialists. Cross‐disciplinary collaboration is key to ensuring algorithm design reflects real‐world variation, clinical context, and professional standards [9, 10, 11]. The radiographer's role as the first point of contact in image acquisition places them at the centre of this transformation, and their involvement must not be peripheral, but central.

There is also a clear opportunity to incorporate AI into radiography education and CPD pathways. A European survey of over 2000 radiographers found widespread agreement that AI education is currently lacking and must be prioritised to ensure future professionals are equipped to handle evolving roles [12]. Future radiographers will be expected to interpret algorithmic outputs, understand AI limitations, and make informed decisions about when to trust or question the model. Building these competencies early in training ensures that AI is seen not as a threat, but as a tool that strengthens professional judgment.

As AI tools become more widely implemented, it is essential to address potential sources of bias arising from differences in patient demographics, imaging equipment, and acquisition protocols. Bias mitigation strategies such as using multi‐institutional datasets, incorporating vendor diversity, and routine algorithm auditing should form part of every AI quality assurance framework. Embedding these practices within radiography governance structures will ensure equitable and generalisable outcomes across patient populations.

From a research perspective, AI studies should continue to explore diverse datasets, including cases from different healthcare settings, patient populations, and imaging equipment. Furthermore, future work may benefit from incorporating clinical metadata to contextualise decision‐making, an aspect the authors rightly noted as a limitation in their study. Ultimately, the goal is not to automate radiographers out of the picture, but to empower them with tools that enhance efficiency, safety, and consistency so that their expertise is amplified, not replaced. In low‐resource and after‐hours settings, AI‐enabled quality checks can reduce variability and unnecessary repeats, supporting safer, more consistent imaging.

## 5 Conclusion

Radiographers are pivotal to ensuring that AI for radiographic image quality enhances safety, consistency, and patient outcomes. The featured study illustrates how AI can serve as a partner in reducing unnecessary exposures and optimising workflow, not merely as a diagnostic aid. Realising this potential requires (i) radiographer leadership in development, validation, and implementation; (ii) sustained education and continuing professional development; and (iii) collaboration with radiologists, medical physicists, and AI specialists. At the same time, adoption must align with practical constraints such as technical infrastructure, regulatory compliance, and interdisciplinary coordination. By embracing innovation while navigating these barriers, radiographers can ensure that AI integration enhances safe and patient‐centred care rather than disrupts it.

## Conflicts of Interest

The author serves as an Associate Editor for the Journal of Medical Radiation Sciences but had no involvement in the editorial decision‐making for this manuscript.

## Data Availability

Data sharing not applicable to this article as no datasets were generated or analysed during the current study.
